# The relationship between parental acceptance-rejection perception and individuals’ levels of emotional intelligence, emotional coping, and family life satisfaction

**DOI:** 10.1186/s41155-026-00403-3

**Published:** 2026-06-13

**Authors:** Şükriye Yeniçeri, Tülay Yılmaz Bingöl

**Affiliations:** 1 Psychiatry Nursing, Tokat Gaziosmanpaşa University, Postgraduate Education Institute, Tokat, Türkiye; 2 Faculty of Health Sciences, Department of Psychiatric Nursing, Tokat Gaziosmanpaşa University, Tokat, Türkiye

**Keywords:** Parental Acceptance-Rejection, Emotional Intelligence, Emotional Coping, Family Life Satisfaction

## Abstract

**Aim:**

This study was conducted to determine the relationship between parental acceptance-rejection theory and emotional intelligence, emotional coping, and family life satisfaction levels in individuals.

**Method:**

This descriptive and correlational study was conducted between 01.11.2021 and 01.04.2022 using the online snowball sampling method via the Google platform with 750 individuals aged 18 and over who agreed to participate. The data were collected using the Personal Data Form, Parental Acceptance-Rejection Scale (PARS), Emotional Intelligence Scale (EIS), Emotional Approach Coping Scale (EACS), and Family Life Satisfaction Scale (FLSS).

**Findings:**

According to the results of the study, the mean PARS score was 39.17 ± 12.06, the mean EIS score was 71.12 ± 15.76, the mean EACS score was 56.97 ± 14.26 and the mean FLSS score was 45.45 ± 22.14. A significant and negative correlation was found between the PARS and the EIS and EACS, while a significant and positive correlation was observed with the FLSS. A significant and positive correlation was found between the EIS and the EACS, while a significant and negative correlation was found between the EACS and the FLSS. Furthermore, a significant and negative correlation was also found between the EACS and the FLSS. Regression analyses revealed that the PARS score was significantly predicted by gender, having children, income level, consultation with a psychological professional, and a history of psychological treatment within the family. Having children and consultation with a psychological professional increased the score, while male gender and high income had a decreasing effect. The EIS score was significantly predicted only by the mother’s living situation, the EACS score by the father’s living situation, and the FLSS score by marital status and consultation with a psychological professional. These findings summarize the explanatory power of the relationships between the psychosocial variables central to the study and parental perception, emotional intelligence, and family life satisfaction.

**Conclusion:**

In our study, it was observed that those who experienced parental rejection had lower emotional intelligence and emotional coping scores and higher family life scores. The results confirmed our assumption and supported Rohner’s parental acceptance-rejection theory.

## Introduction

Parental acceptance-rejection theory is a lifelong development and socialization theory that examines the causes, consequences, and relationships with other variables of parental acceptance and rejection in the developmental process of individuals from birth to death, (Rohner, 2004, 2016). Parental Acceptance-Rejection Theory, which adopts a phenomenological approach to the parent-child relationship, is not a specific behavior exhibited by parents, but the child’s belief in this behavior (Rohner & Lansford, 2017).

The earliest childhood relationships with a primary caregiver have a major impact on the entire life. Attachment patterns formed in early childhood shape individuals’ personal and social lives, professional relationships, stress regulation processes, and cognitive development. Moreover, these early relational experiences continue to influence both physical and mental health across the lifespan (Štrosar et al., 2024).

In parental acceptance-rejection theory, the presence of love and affection by parents towards their children is defined as parental acceptance, and the absence of love and affection is defined as parental rejection (Rohner and Khaleque 2002a, b). In the theory, acceptance and rejection by parents constitute the warmth dimension of parenting and are related to the quality of the emotional bond in the parent-child relationship and the verbal and nonverbal behaviors they use to express emotions. Acceptance is at the positive end of the dimension, and rejection is at the negative end. Acceptance is the interest, compassion, warmth and closeness that parents show towards their children while rejection is the attitude where emotions and behaviors are absent or clearly withheld, and physical and/or psychological behaviors and/or feelings that hurt the child are exhibited (Can & Aksel, 2017).

Mothers and fathers show rejection behavior towards their children in four ways (Bircan & Erden, 2011): Parental warmth/affection (or coldness/lack of love), Hostility/aggression, Indifference/neglect and Undifferentiated rejection (Rohner & Khaleque, 2010; Rohner et al., 2020). The first three include parents’ cold, aggressive and neglectful behaviors towards their child. Undifferentiated rejection is when children feel that their mothers or fathers do not truly love, care about, appreciate them or otherwise devalue them, without objective indications that the parents are aggressive, neglectful or emotionally/physically hurtful (Haktanır & Çoklar, 2023; Rohner, 2004, 2005; Rothenberg et al., 2022).

Emotional intelligence emerges as an important concept in understanding individuals’ emotions and coping effectively in this process. Salovey and Mayer defined emotional intelligence as a subset of social intelligence that includes the capacity to monitor one’s own and others’ feelings and emotions, to discriminate between them, and to use this information to manage one’s thoughts and actions (Salovey & Mayer, 1990). An individual with high emotional intelligence is someone who is aware of his/her strengths and weaknesses, knows himself/herself and his/her needs, manages to control his/her emotions, and establishes effective relationships. Individuals with high emotional intelligence are more successful in problem solving (Tetik & Açıkgöz, 2013).

Parental acceptance was reported to be associated with effective coping with stressful life events (with problem-focused coping) while parental rejection was mentioned to be associated with more emotion-focused coping. It was observed that warm and open communication between the mother and the child develops a more constructive coping in the child (Moran et al., 2018). One of the important issues that parental acceptance rejection is associated with is coping. Emotional coping includes behaviors aimed at controlling the negative emotions created by the stress situation and focusing on a positive aspect (Eryılmaz & Ercan, 2010). When individuals experience stress, they may use avoidance or distancing to reduce emotional response. They may try to motivate themselves by focusing on the positive aspects of the event or increasing negative thoughts about the event in order to better manage the stressful situation (Stanton et al., 1994).

Family life satisfaction includes the thoughts of family members on their interactions with each other and the perception of certain aspects of family members and family life. Parental acceptance-rejection affects family life satisfaction. Family life satisfaction is an individual characteristic that may differ from family to family rather than a general concept (Barraca et al., 2000). Since an individual who perceives acceptance has a higher family life satisfaction, their perspective on events and problem-solving approach would be more effective.

Parents’ acceptance attitudes towards their children have profound effects on emotional intelligence, emotional coping and family life satisfaction in children. This relationship is critical in individuals’ healthy psychological and emotional development. Although there are many studies conducted on different age groups and various variables regarding parental acceptance rejection theory, there is no study on the emergence of possible problems with the use of awareness and effective coping methods, which constitute the cornerstones of mental health. In this study, which aims to fill this gap in the literature, it is aimed to determine the relationship between parental acceptance-rejection theory and emotional intelligence, emotional coping and family life satisfaction levels in individuals.

### Research hypotheses

- H1.1: There is a significant relationship between perceived parental acceptance/rejection and the level of emotional intelligence in adulthood.
- H1.0: There is no significant relationship between perceived parental acceptance/rejection and the level of emotional intelligence in adulthood.
- H2.1: There is a significant relationship between perceived parental acceptance/rejection and the level of emotional coping in adulthood.
- H2.0: There is no significant relationship between perceived parental acceptance/rejection and the level of emotional coping in adulthood.
- H3.1: There is a significant relationship between perceived parental acceptance/rejection and the level of family life satisfaction in adulthood.
- H3.0: There is no significant relationship between perceived parental acceptance/rejection and the level of family life satisfaction in adulthood.

### Type of research

It is a descriptive and correlational type research.

### Research universe and sample

The research sample consisted of all adults who were 18 years of age and over, who were literate and agreed to participate in the research between the dates of 01.11.2021 and 01.04.2022, via the Google platform when the research was conducted, using the online snowball method. The number of people to be included in the sample was calculated by the G*Power software using the data from the study titled ‘The Relationship between Parental Acceptance-Rejection and Parental Practices and the Influencing Factors’ by Can and Aksel (2017). The sample size of the study was planned to be 462 individuals with 99% power, 5% margin of error and 0.40 effect size calculated based on the t-test analysis in the G*Power 3.1.9.2 statistical software. To increase the reliability and representativeness of the findings, the aim was to obtain a larger sample size. The study included 780 individuals. However, the final number of the subjects was 750 because 22 of the individuals were under the age of 18 and 8 of them filled out the scales incompletely. No data completion (imputation) method was applied for missing data, and the missing data rate was 1.03% of the total sample. All statistical analyses were performed only on the complete dataset. The average age of the participants was 26.75 ± 8.81 years. It was determined that 73.5% of the individuals were female and 64.5% were single. Ethical permission was obtained before starting this study conducted in Turkey (October 1, 2021, Session 20, Decision No. 12). The principle of voluntary participation was applied during the study process and the participants were informed with an informed consent form. In the study, which proceeded with the online snowball method, different age groups and different social environments were used in order not to limit the generalizability of the study with a limited sample.

This study was conducted using a cross-sectional and correlational design, and data were collected through self-report scales. This approach carries the risk of social desirability and recall bias. Furthermore, due to the cross-sectional design, causal inferences between variables cannot be made. To reduce potential biases, data collection was conducted anonymously; no identifying information was requested from participants, and validated and reliable measurement instruments were used.

**Figure:**
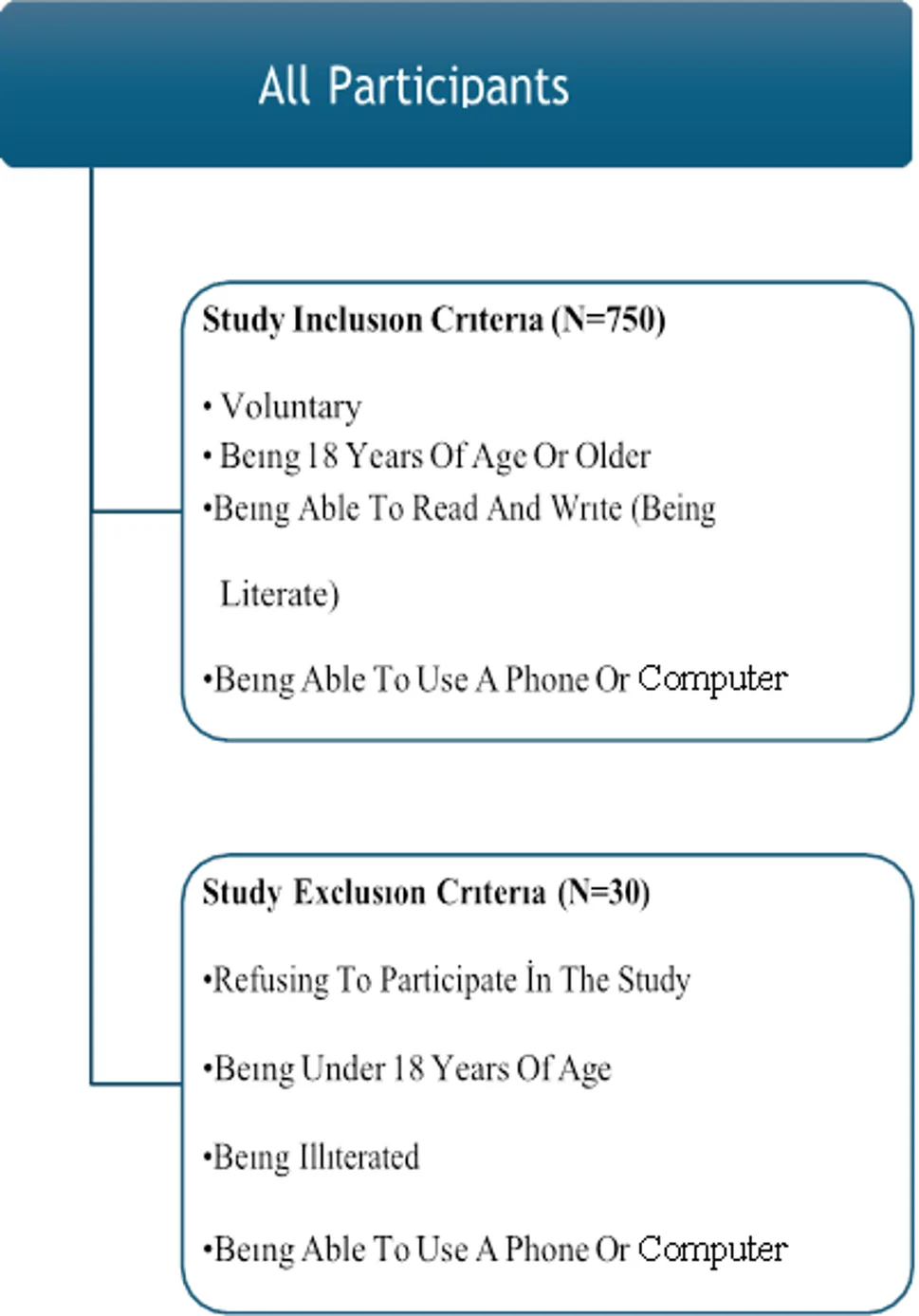

### Data collection tools

#### Personal information form

The personal information form was prepared by the researcher as a result of the literature review. This form contains a total of 17 questions including sociodemographic data of individuals such as gender, marital status, education level, etc. and is intended to evaluate several important parameters in terms of parental acceptance-rejection (Kilisli, 2015; Özbaş, 2010).

#### Parental acceptance-rejection scale (PARS)

The Parental Acceptance-Rejection Scale was developed by Ronald Rohner (1986) to assess individuals’ perceptions of parental acceptance-rejection within four frameworks and was adapted to Turkish by Dedeler et al. (2017). It has three versions: adult, child and parent. The adult version assesses adults’ perceptions of their parents’ behavior towards them when they were approximately seven to twelve years old. The tool consists of Mother and Father Forms having exactly the same items. The scale assesses perceived parental acceptance-rejection across four dimensions: warmth/affection, hostility/aggression, indifference/neglect and undifferentiated rejection. The scale, consisting of twenty-four items, has four sub-dimensions: warmth/affection (eight items), hostility/aggression (six items), indifference/neglect (six items) and undifferentiated rejection (four items). Each item is evaluated on a 4-point Likert-type scale and scored as “almost always true” (4 points), “sometimes true” (3 points), “rarely true” (2 points) and “almost never true” (1 point). The scores of the subscales are obtained by summing the scores of their items. The score obtained from the scale varies between 24 (highest level of acceptance) and 96 (highest level of rejection), and the internal consistency coefficients of the subscales of the original scale were reported to be over 0.81 (Dedeler et al., 2017). In our study, the Cronbach’s Alpha coefficient of the scale was 0.94, and subscale coefficients were 0.90 for warmth-affection, 0.85 for hostility-aggression, 0.83 for indifference-neglect and 0.87 for undifferentiated rejection.

#### Emotional intelligence scale (EIS)

The scale, which was developed by Hyuneung Lee and Yungjung Kwak in South Korea in 2012, was adapted to Turkish by Kayıhan and Arslan (2016). The scale consists of three sub-dimensions as “Emotional Recognition”, “Emotional Facilitation”, and “Emotional Regulation”. There are 20 items on the 5-point Likert-type scale. The scale is scored from Strongly Disagree (1 points) to Strongly Agree (5 points). The lowest score that can be obtained from the scale is 20, and the highest is 100. High average total scores represent high emotional intelligence levels. Cronbach’s Alpha reliability coefficient was 0.83 for the whole scale, 0.72 for emotional recognition sub-dimension, 0.71 for emotional facilitation sub-dimension and 0.76 for emotional regulation sub-dimension (Kayıhan & Arslan, 2016). In our study, Cronbach’s Alpha coefficient for the whole scale was 0.94 while subscale coefficients were 0.87 for emotional recognition, 0.81 for emotional facilitation and 0.91 for emotional regulation.

#### Emotional approach coping scale (EACS)

The scale, developed by Stanton et al. (2000) and adapted to Turkish by Şenol-Durak and Durak (2011), is used to assess emotional coping. It is a self-report scale consisting of a total of 16 items evaluated on a 5-point Likert-type, and has two subscales (emotional processing and emotional expression). The Cronbach’s Alpha coefficient is 0.91 for the “Emotional expression” subscale and 0.89 for the “Emotional processing” subscale while the Cronbach’s Alpha coefficient for the total score is 0.89 (Şenol-Durak & Durak, 2011). In our study, Cronbach’s Alpha coefficient was 0.96 for the total score, 0.94 for the emotional expression subscale, and 0.93 for the emotional processing subscale.

#### Family life satisfaction scale (FLSS)

The scale is a Likert-type scale consisting of 23 items developed by Çalışkan et al. in 2017 to determine the level of satisfaction of family members in the family life environment. The scale items consist of opposite adjectives related to perceived life satisfaction in the family, located at the right and left ends of the item. The scoring of the items is evaluated with an increasing value from negative to positive expression. Thus, it is scored from negative to positive expression (1. Strongly disagree, 2. Disagree, 3. Slightly disagree, 4. Neither agree nor disagree, 5. Slightly agree, 6. Agree and 7. Strongly agree). The lowest score that can be obtained from the scale is 23, the highest score is 161. Higher scores indicate that the individual has higher life satisfaction when he/she is with his/her family. The Cronbach α coefficient of the scale was 0.95 (Çalışkan et al., 2017). In our study, the total internal consistency coefficient of the scale was 0.98.

### Limitations

The most significant limitation of the study is that, although data were collected using an online snowball method, the views of some communities may be overlooked or misinterpreted due to demographic groups with limited internet access and digital literacy. Therefore, care was taken to ensure that the surveys were simple and understandable. To avoid privacy concerns, individuals were informed that the collected data was for research purposes only. Furthermore, while descriptive and correlational design reveals relationships between variables, it may not allow for causal inferences. Data collection through self-report scales may carry the risk of social desirability and recall bias. The study functionalizes broad theoretical concepts through specific sub-dimensions of the scale, and findings should only be interpreted within the framework of the measured empirical dimensions.

### Data analysis

The data from the applied personal data form, Parental Acceptance-Rejection Questionnaire, Emotional Intelligence Scale, Emotional Approach Coping Scale and Family Life Satisfaction Scale were analyzed using the IBM SPSS Statistics 22, SPSS Inc., an IBM Co., Somers, NY statistical software, and evaluated based on the relevant literature (George & Malley, 2021). Descriptive statistics were used to provide information about the general characteristics of the study. Data for quantitative variables were defined as mean and standard deviation (X ± SD) whereas data for qualitative variables were defined as frequencies and percentages (n(%). Correlation and regression analyses were used in the calculations of direction, magnitude and explanatory power of the relationships between variables. In the statistical tests performed, 95% confidence interval and *p* < 0.05 were taken as the significance level.

## Results

Table 1 shows the distribution of individuals according to their descriptive characteristics. The average age of the individuals examined in the study was 26.75 ± 8.81 years. It was determined that 73.5% of the individuals were female, 64.5% were single, 69.1% did not have children, 55.5% had college degree, 78% evaluated their income at a moderate level, 95.1% had a living mother and 89.6% had a living father, 95.3% had parents living together when they were children, 57.5% had a mother and 39.7% had a father who was a primary school graduate, 72.7% had a mother who did not work during their childhood, 80.7% did not see an expert for psychological reasons, and 82.3% did not have anyone in their family who received psychological treatment.

**Table 1:** Descriptive Characteristics of the Study Participants

Age	Ort	SS	Me	Min	Mak
	26,	8,81	23,	15,0	72,0
			**n**	**%**	
Gender	Male		199	26.5	
	Female		551	73.5	
Marital status	Single		484	64.5	
	Married		266	35.5	
Having child	No		518	69.1	
	Yes		232	30.9	
Education status	Primary school		27	3.6	
	High school		90	12.0	
	Two-year college		181	24.1	
	Undergraduate or graduate		416	55.5	
	Other		36	4.8	
Income level	Low		133	17.7	
	Moderate		585	78.0	
	High		32	4.3	
Mother living or deceased	Deceased		37	4.9	
	Living		713	95.1	
Father living or deceased	Deceased		78	10.4	
	Living		672	89.6	
Were parents together during childhood?	Divorced		21	2.8	
	Married, living separate ayrı yaşıyorlar		14	1.9	
	Married, living together		715	95.3	
Education status of mother	Illiterate		108	14.4	
	Primary school		431	57.5	
	Secondary school		92	12.3	
	High school		83	11.1	
	College		36	4.8	
Education status of father	Illiterate		29	3.9	
	Primary school		298	39.7	
	Secondary school		112	14.9	
	High school		181	24.1	
	College		130	17.3	
Mother’s working status during childhood	No		545	72.7	
	Yes		205	27.3	
Applying to a psychological expert	No		605	80.7	
	Yes		145	19.3	
Anyone receiving physiological treatment in family	No		617	82.3	
	Yes		133	17.7	

It was observed that the individuals’ total mean score of the PARS was 39.17 ± 12.06 (Min = 26 - Max = 92), the mean score of “Warmth-Affection” was 26.54 ± 5.42 (Min = 8 - Max = 32), the mean score of “Hostility-Aggression” was 9.20 ± 3.87 (Min = 6 - Max = 24), the mean score of “Indifference-Neglect” was 9.50 ± 3.66 (Min = 6 - Max = 24), the mean score of “Undifferentiated Rejection” was 5.48 ± 2.56 (Min = 4 - Max = 16). The individuals’ total mean score of the EIS” was 71.12 ± 15.76 (Min = 20 - Max = 100), the mean score of “Emotional Recognition” was 22.58 ± 5.25 (Min = 6 - Max = 30), the mean score of “Emotional Facilitation” was 21.06 ± 5.12 (Min = 6 - Max = 30) and the mean score of “Emotional Regulation” was 27.47 ± 7.42 (Min = 8; Max = 40). It was found that the mean score of the individuals in the EACS was 56.97 ± 14.26 (Min = 16 - Max = 80), the mean score of “Emotional Expression” was 27.71 ± 7.55 (Min = 8 - Max = 40), and the mean score of “Emotional Processing” was 29.26 ± 7.35 (Min = 8 - Max = 40). It was observed that the total mean score of the individuals on the FLSS was 45.45 ± 22.14 (Min = 23 - Max = 159). (Table 2).

**Table 2:** Participants’ Mean Scores for the Scales

		Number of items	Cronbach’s Alpha	Ort	SS	Medyan	Min	Max
Parental Acceptance-Rejection Scale (PARS)	Warmth/affection	8	0.919	26.54	5.42	28.00	8.00	32.00
	Hostility/aggression	6	0.856	9.20	3.87	8.00	6.00	24.00
	Indifference/neglect	6	0.834	9.50	3.66	8.00	6.00	24.00
	Undifferentiated rejection	4	0.877	5.48	2.56	4.00	4.00	16.00
	Total	24	0.942	39.17	12.06	35.00	26.00	92.00
Emotional Intelligence Scale (EIS)	Emotional Recognition	6	0.886	22.58	5.25	23.00	6.00	30.00
	Emotional Facilitation	6	0.807	21.06	5.12	22.00	6.00	30.00
	Emotional Regulation	8	0.913	27.47	7.42	29.00	8.00	40.00
	Total	20	0.939	71.12	15.76	74.00	20.00	100.00
Emotional Approach Coping Scale (EACS)	Emotional expression	8	0.937	27.71	7.55	29.00	8.00	40.00
	Emotional processing	8	0.951	29.26	7.35	31.00	8.00	40.00
	Total	16	0.965	56.97	14.26	60.00	16.00	80.00
Family Life Satisfaction Scale (FLSS)		23	0.979	45.45	22.14	41.00	23.00	159.00

**Fig. 1:**
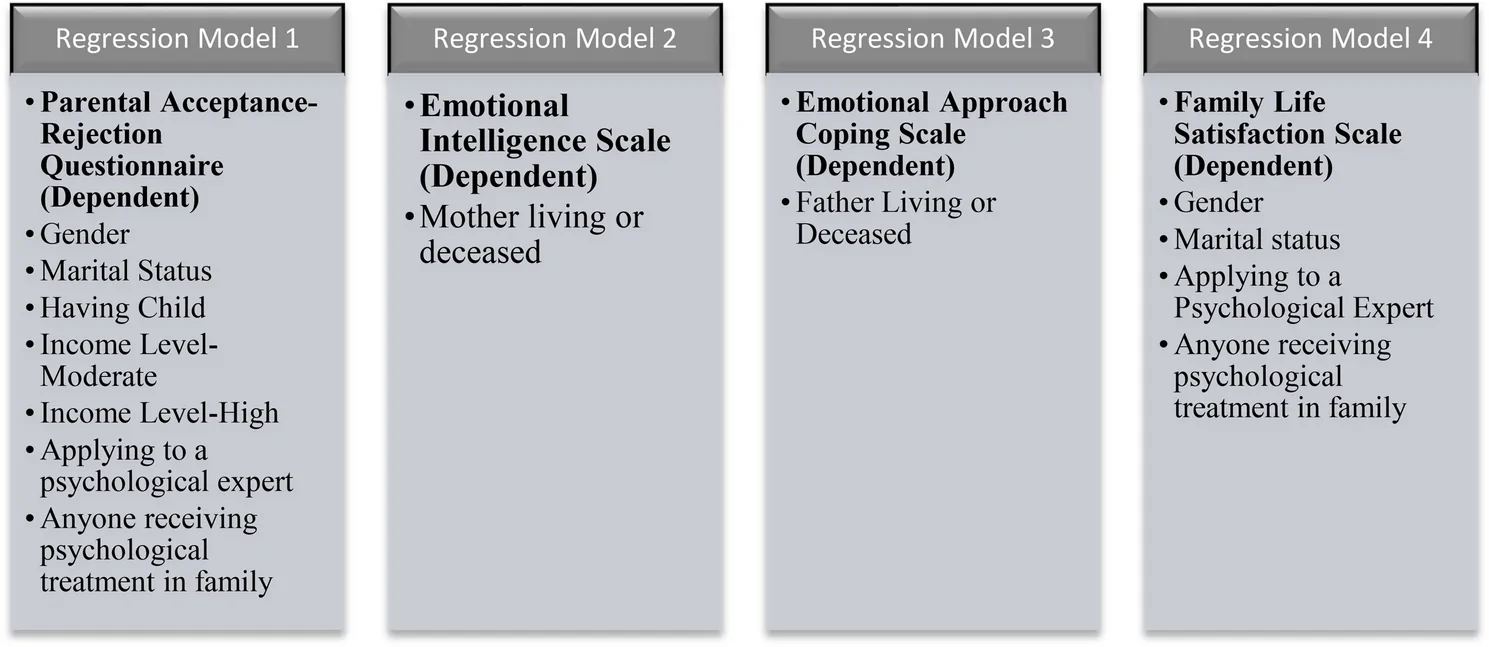
Independent and Dependent Variables for The Regression Models Created

Table 3 shows the correlations between the tools used in our study and their sub-dimensions. As a result of the correlation analysis, it was revealed that there was a significant, negative, weak relationship between PARS score and EIS score (*r*=-0.26; *p* < 0.001), a significant, negative but weak relationship between PARS score and EACS score (*r*=-0.25; *p* = 0.002), a significant, positive and moderate relationship between PARS score and FLSS score (*r* = 0.50; *p* < 0.001). The relationship between EIS score and EACS score (*r* = 0.62; *p* < 0.001) was significant, positive and moderate; and the relationship between EIS score and FLSS score was significant, positive and weak (*r* = 0.133; *p* < 0.001). It was revealed that there was a significant, negative and weak relationship between EACS score and FLSS score (*r*=-0.15; *p* < 0.001). (Figure 1).

**Table 3:** Relations Between PARS, EIS EACS, and FLSS

		EIS	EACS	FLSS
PARS	r	-0.263	-0.257	0.503
	p	< 0.001*	0.002*	< 0.001*
EIS	r		0.627	-0.133
	p		< 0.001*	< 0.001*
EACS	r			-0.155
	p			< 0.001*

*r* Pearson correlation coefficient

*significant at 0.05 level

Multiple linear regression analyses were conducted to identify the predictors of Parental Acceptance-Rejection (PARS), Emotional Intelligence (EI), Emotional Coping (EACS), and Family Life Satisfaction (FLSS). Independent variables that demonstrated significant associations in preliminary analyses were entered simultaneously into the regression models. Standardized beta coefficients (β), unstandardized coefficients (B), and significance levels (p) were reported. (Table 4).

**Table 4:** Multiple Regression Analysis of the Relationship Between PARS and Other Variables

Independent variables of the model	Non-standardized coefficients		Standardized Beta	t	*p*	Confidence interval for B (95%)	
	B	Std. error				Lower limit	Upper limit
Model 1 Dependent variable: PARS							
Gender	-2.974	0.971	-0.109	-3.065	0.002*	-4.88	-1.069
Having child	5.055	1.691	0.194	2.99	0.003*	1.736	8.375
Income level - Moderate	-2.732	1.123	-0.094	-2.434	0.015*	-4.936	-0.528
Income level - High	-7.288	2.329	-0.122	-3.128	0.002*	-11.861	-2.715
Applying to a psychological expert	5.363	1.105	0.176	4.852	< 0.001*	3.193	7.533
Anyone receiving psychological treatment in family	4.188	1.135	0.133	3.691	< 0.001*	1.96	6.416
*R* = 0.322; R^2^ = 0.103; Adjusted R^2^ = 0.095			F:12.224; *p* < 0.001*				
Model 2 Dependent variable: EIS							
Mother living or deceased	5.244	2.652	0.072	1.977	0.048*	0.037	10.450
*R* = 0.072; R^2^ = 0.005			F:3.909; *p* = 0.048*				
Model 3 Dependent variable: EACS							
Father living or deceased	3.501	1.702	0.075	2.057	0.040*	0.161	6.842
*R* = 0.075; R^2^ = 0.006			F:4.233; *p* = 0.040*				
Model-4 Dependent variable: FLSS							
Marital status	-3.758	1.64	-0.081	-2.292	0.022*	-6.978	-0.539
Applying to a psychological expert	13.364	2.051	0.239	6.516	< 0.001*	9.338	17.39
*R* = 0.281; R^2^ = 0.079; Adjusted R^2^ = 0.074			F:15.984; *p* < 0.001*				

*significant at 0.05 level

The regression model predicting PARS scores was statistically significant (*p* < 0.05). Gender (β = −0.109, *p* < 0.05), having children (β = 0.194, *p* < 0.05), medium income level (β = −0.094, *p* < 0.05), high income level (β = −0.122, *p* < 0.05), application to a psychological specialist (β = 0.176, *p* < 0.05), and presence of psychological treatment in the family (β = 0.133, *p* < 0.05) emerged as significant predictors.

Specifically, gender was negatively associated with PARS scores, while having children and applying to a psychological specialist were positively associated with higher PARS scores. Similarly, having a family member who received psychological treatment was positively related to PARS. In contrast, medium and high income levels were negatively associated with PARS scores compared to the reference income category. These findings indicate that both sociodemographic characteristics and psychological support experiences are associated with perceived parental acceptance–rejection.

The regression model for Emotional Intelligence was statistically significant (*p* < 0.05). Mother’s living status significantly predicted EI scores (β = 0.072, *p* < 0.05). Participants whose mothers were alive reported higher emotional intelligence levels. Although the effect size was modest, this finding suggests that maternal presence may have a lasting association with emotional development.

The regression model predicting Emotional Coping (EACS) was statistically significant (*p* < 0.05). Father’s living status significantly predicted EACS scores (β = 0.075, *p* < 0.05). Participants whose fathers were alive demonstrated higher emotional coping levels. This result suggests that paternal presence may contribute to the development of emotional coping capacities.

The regression model for Family Life Satisfaction was also statistically significant (*p* < 0.05). Marital status (β = −0.081, *p* < 0.05) and application to a psychological specialist (β = 0.077, *p* < 0.05) significantly predicted FLSS scores. Marital status was negatively associated with family life satisfaction, whereas seeking psychological support was positively associated with FLSS. These findings indicate that relational status and help-seeking behaviors are relevant factors in explaining variations in perceived family life satisfaction.

## Discussion

This study aimed to examine the relationship between parents’ perception of acceptance and rejection and individuals’ emotional intelligence, emotional coping, and family life satisfaction. This section discusses the findings obtained from the analyses conducted in accordance with the study’s objective, in light of the literature.

In this study, a significant relationship was found between perceived parental acceptance and rejection and emotional intelligence level in adulthood; that is, those who perceived parental acceptance and rejection had higher levels of emotional intelligence, while those who perceived rejection had lower levels of emotional intelligence. This finding is consistent with our H1.1 hypothesis and suggests that perceived parental acceptance/rejection may play a role in emotional intelligence in adulthood. In a study conducted by Rohner (2003) with adolescents who experienced acceptance-rejection by their parents in their childhood, it was found that the emotional empathy levels of girls were related to the acceptance of the mother, while those of boys were related to the acceptance of the father. Girls who perceived themselves to be rejected by their mothers had more negative levels of emotional empathy with their mothers than girls who perceived themselves to be accepted by their mothers. In a similar study conducted on the role of parental acceptance-rejection in emotional instability during adolescence, a clear relationship was found between emotional instability and mother/father criticism and rejection, and especially mother criticism and rejection in early adolescence and father criticism and rejection in mid-adolescence were associated with emotional instability (Mendo-Lázaro et al., 2019). The study found a statistically significant difference in emotional intelligence between boys and girls based on their fathers’ acceptance of them; girls who felt their fathers approved of them scored higher on the emotional intelligence variable (Chohan & Habib, 2024). Another study have shown that emotional intelligence in adolescents is significantly related to their perception of paternal acceptance or rejection, and that parental acceptance is also a significant indicator of emotional intelligence in adolescents (Hafeez & Habib, 2021). These studies, which examined parental acceptance-rejection and emotional approach, lend support our study. Individuals who are accepted by their parents can express themselves more easily, strengthen their communication with the others and maintain their emotional balance. On the other hand, in a study examining the relationship between the parental acceptance-rejection that the child perceives from his/her mother and father and the child’s emotion regulation skills, no relationship was found between the acceptance and control that the child perceives from both his/her mother and father and the emotion regulation skills (Gelgör, 2016). No study was found in the literature with the same scales and samples to compare our research results. Although it is difficult to discuss these findings due to the limited findings in the literature, while a significant relationship between parental acceptance-rejection and EI and its sub-scales cannot always be determined, it can be assumed that this result was affected by factors such as different cultures, environments, and upbringing conditions of the study group. However, the EI level of individuals can be improved by relevant educational institutions during the education period and by controls and regular training that can be organized by managers in business life.

Our study found a significant relationship between perceived parental acceptance and rejection and the level of emotional coping in adulthood; individuals who felt accepted by their parents had better emotional coping skills, while those who perceived themselves as rejected showed lower emotional coping abilities. This finding supports our hypothesis H2.1 and suggests that perceived parental acceptance/rejection may have a significant impact on emotional coping in adulthood. In Yılmaz’s study, it was observed that students who established warm and loving relationships with their parents and thought that their parents respected their decisions were also good at problem solving, communication skills, coping with stress, and skills that increased self-esteem (Yılmaz, 2014). It was observed that mothers’ parenting practices were especially important on adolescent drug addiction, and since addicts perceived their mothers as more rejecting than non-addicts, mothers’ rejection could be one of the main risk factors for developing drug addiction (Glavak et al., 2003). In a similar study, it was determined that parents’ emotional warmth had a positive effect on adolescent mental health through the chain mediation effects of self-esteem and psychological flexibility. It can be stated that parental rejection and parental overprotection have negative effects on adolescent mental health by decreasing self-esteem but increasing psychological flexibility (Peng et al., 2021). In a study examining the relationship between parental acceptance-rejection perception and stress coping styles among college students, a positive relationship was found between the sub-dimensions of the maternal acceptance/rejection scale, “warmth/affection” and self-confident approach and a negative relationship was found between hostility/aggression, indifference/neglect, and undifferentiated rejection and self-confident approach (Hasanova, 2020). Due to the limited studies in the literature, no similar study has been found on this subject with adults. Studies conducted with different age groups, as mentioned above, have shown that individuals rejected by their families are less likely to use effective coping mechanisms, are more likely to feel worthless and use substances, and are more likely to experience problems with problem-solving and communication skills. Therefore, the literature supports our study.

In the present study, the relationship between emotional intelligence and emotional coping was found to be significant and as the level of emotional intelligence increases, the individual’s possibility of emotional coping also increases. In İşmen’s study, it was observed that as the level of emotional intelligence increased, the perception of problem-solving skills developed (İşmen, 2001). Similarly, in the study conducted by Karabulutlu et al. on students, it was observed that as the level of emotional intelligence of the students increased, their problem-solving skills were elevated (Karabulutlu et al., 2011). In the study conducted by Deniz and Yılmaz on university students, a positive and significant relationship emerged between the stress coping dimension of emotional intelligence and the problem-focused coping style among stress coping styles (Deniz & Yılmaz, 2006). In the study conducted by Özer and Deniz, a positive and significant relationship was found between the psychological resilience scores and emotional intelligence sub-dimension scores of university students (Özer & Deniz, 2014). In a study investigating the relationship between emotional intelligence and burnout in healthcare workers, it was shown that the four dimensions of emotional intelligence were negatively related to the desensitization dimension of burnout and positively related to the personal accomplishment dimension. In addition, the positive emotional management and empathic sensitivity dimensions of emotional intelligence were reported to have a negative relationship with the emotional exhaustion dimension of burnout, that is, an increase in the level of emotional intelligence increased the personal success dimension of burnout, while a decrease in the level of emotional intelligence could decrease personal success (Arslan & Özata, 2008). In the study conducted by Tetik and Açıkgöz (2013) investigating the effect of emotional intelligence level on problem-solving skills, an increase in problem-solving skills was observed as the emotional intelligence level of individuals increased (Tetik & Açıkgöz, 2013). In a study investigating the dimensions of emotional intelligence as a predictor of coping responses to stress in nursing practitioners, it was shown that the evaluation of others’ emotions was a predictor of self-blame and the use of emotions was a predictor of positive reframing and self-blame (Ali et al., 2019). No study was found in the literature using the same scales and samples to compare our research results. Although it was difficult to discuss these findings due to the limited studies in the literature, according to existing studies, it was observed that as emotional intelligence increases, individuals’ emotional awareness and empathy increase, which helps them establish healthier relationships and cope with stress more effectively, and as emotional intelligence decreases, psychological flexibility decreases, emotional reactions become excessive, and it becomes difficult to cope with stressful situations. Thus, the literature on different sample groups and variables supports our study.

In the present study, the relationship between emotional intelligence and family life satisfaction was significant and as emotional intelligence increases, family life satisfaction is positively affected. In Özdemir and Dilekmen’s study, a positive significant relationship was found between life satisfaction and emotional intelligence total and sub-dimensions. In addition, emotional intelligence was found to be a significant predictor of life satisfaction (Özdemir & Dilekmen, 2016). While no similar study conducted with adults can be found in the literature, in the study conducted by Çetintaş (2021), which investigated the relationship between family life satisfaction and psychological resilience and emotional autonomy in adolescents, it was observed that as the family life satisfaction levels of adolescents increased, their emotional autonomy decreased and their psychological resilience increased (Çetintaş, 2021). In the study conducted by Kılınç (2021), which investigated the relationship between social media addiction, family life satisfaction and psychological well-being in adolescents, it was determined that family life satisfaction had a strong effect on increasing the psychological well-being of adolescents, and activities that would increase life satisfaction within the family reduced social media addiction and increased psychological well-being (Kılınç, 2021). The literature on different sample groups and variables supports our study.

In our study, it was revealed that this issue could be evaluated in a wider universe and sample level. The issue could be re-examined with a universe that would cover the whole of Türkiye using a more diverse and larger sample.

Policy and practice can be guided by topics such as awareness programs for families, parenting education, psychoeducation and counseling services, stress management that provides emotional support for children, school-based interventions focused on problem-solving and effective communication, preventive interventions for at-risk groups, and public awareness campaigns. To ensure the continuity of training and verify its effectiveness while working on these topics, the process should be supported by regular checks, evaluation forms, or projects.

## Conclusion and suggestions

This study investigated the relationships between perceived parental acceptance/rejection and individuals’ levels of emotional intelligence, emotional coping, and family life satisfaction. The findings revealed that parental acceptance/rejection is a significant predictor of both emotional intelligence and emotional coping. Individuals who perceived themselves as accepted by their parents during childhood were found to have higher levels of emotional intelligence and more effective emotional coping skills; those who perceived themselves as rejected exhibited lower levels in these areas. Furthermore, a positive and significant relationship was found between emotional intelligence and emotional coping; as emotional intelligence levels increased, individuals’ likelihood of coping with stress and emotional difficulties also increased. In addition, a positive and significant relationship was found between emotional intelligence and family life satisfaction, indicating that emotional intelligence is an important variable supporting family life satisfaction. Overall, it can be said that early parent-child relationships have long-term effects on an individual’s emotional development, coping capacity, and satisfaction with family life. The findings largely align with the relevant literature and suggest that parental acceptance may be a protective factor, while parental rejection may be a risk-increasing factor.

Support for parents can be provided through programs such as pregnancy and birth planning, prevention of unwanted pregnancies, psychosocial support for families, strengthening the mother-infant bond during pregnancy and the postpartum period, promoting healthy parent-child communication and interaction, family-focused initiatives to increase parental acceptance, and the dissemination of parenting and psycho-education programs. Performance-oriented interventions focusing on emotional intelligence, problem-solving, and effective communication skills can be implemented in schools; psychosocial support can be provided for at-risk children and adolescents; and special education plans can be developed for children and adults experiencing rejection. Given that healthy parents raise healthy children and healthy children become healthy parents, it is recommended that personal development and awareness programs be organized in institutions serving the community, especially in the fields of health and education.

## Data Availability

The data that support the findings of this study are available on request from the corresponding author. The data are not publicly available due to privacy or ethical restrictions.

## Abbreviations

X±SD: Mean±standard deviation
SPSS: Statistical Package for the Social Sciences
IBM: International Business Machines
n(%): Frequencies and Percentages
PARS: Parental Acceptance-Rejection Scale
EIS: Emotional Intelligence Scale
EACS: Emotional Approach Coping Scale
FLSS: Family Life Satisfaction Scale
